# Risk Stratification for CKD Progression in Primary Care

**DOI:** 10.1016/j.ekir.2025.02.037

**Published:** 2025-03-06

**Authors:** Clyson Mutatiri, Angela Ratsch, Matthew McGrail, Sree Krishna Venuthurupalli, Srinivas Kondalsamy-Chennakesavan

**Affiliations:** 1 Renal Medicine, Bundaberg Base Hospital, Wide Bay Hospital and Health Service, Bundaberg, Queensland, Australia; 2 Rural Clinical School, Faculty of Medicine, The University of Queensland, Bundaberg, Queensland, Australia; 3 Research Services, Wide Bay Hospital and Health Service, Hervey Bay, Queensland, Australia; 4 Rural Clinical School, Faculty of Medicine, The University of Queensland, Hervey Bay, Queensland, Australia; 5 Rural Clinical School, Faculty of Medicine, The University of Queensland, Rockhampton, Queensland, Australia; 6 The University of Queensland, Faculty of Medicine, Brisbane, Australia; 7 Kidney Service, Department of Medicine, West Moreton Hospital and Health Service, Ipswich, Queensland, Australia; 8 Rural Clinical School, Faculty of Medicine, The University of Queensland, Toowoomba, Queensland, Australia; 9 School of Health and Medical Sciences, University of Southern Queensland, Toowoomba, Queensland, Australia

**Keywords:** CKD, eGFR, KFRE, KRT, specialist referral

## Introduction

The kidney failure risk equation (KFRE) was developed in 2011, to quantitate the risk of progression to kidney failure in patents with stage G3 to G5 chronic kidney disease (CKD), and has since been comprehensively applied and validated externally in multinational cohorts.^1^ The equation has been validated in Australia^2^^,^^3^; however, it has not been widely adopted in clinical practice within Australia partly due to lack of formal implementation strategies supporting its routine integration in primary care clinics. Further studies are needed to confirm the clinical impact projections and referral criteria in the Australian context, and our group is involved in the work to address this question.^4^

Australia’s peak body Kidney Health Australia (KHA) currently recommends usage of the estimated glomerular filtration rate (eGFR) thresholds to guide the referral of individuals with CKD from primary care to the nephrologist.^5^ KHA recommends, among other criteria, referral of anyone with an eGFR < 30 ml/min per 1.73 m^2^.

We conducted a retrospective secondary analysis of participants enrolled in the CKD in Queensland-Registry, Australia from a single nephrology service in a referral hospital to evaluate the non-North American 4-variable KFRE as a risk stratification strategy for primary care referrals to nephrology services in comparison to eGFR threshold < 30 ml/min per 1.73 m^2^. The CKD in Queensland Registry enrolled adults aged > 18 years with a diagnosis of CKD, who were attending public renal clinics.S1

## Results

The baseline characteristics of the participants are displayed in Table 1. A total of 1173 participants fulfilled the inclusion criteria for analysis (Supplementary Figure S1), with a median age of 68 years (range: 18–95 years), with more males (55%) than females, and Indigenous participants constituting 10% of the sample. Nearly half of the participants (49%) were diabetic and 92% had hypertension as a comorbidity. The median eGFR at baseline was 33 ml/min per 1.73 m^2^ (interquartile range: 18), and most of the participants (60%) were in stage G3 CKD whereas 5% were in stage G5 CKD at the time of referral to the nephrology clinic. The median urine albumin-to-creatinine ratio was 12mg/mmol (interquartile range: 66.7), and 429 participants (37%) met the KHA albuminuria threshold of > 30 mg/mmol for specialist referral, of whom 61% were diabetic.

**Table 1:** Participants characteristics

Demographics	Participants, *N* = 1173 (%)
Age, mean (SD), yrs	66 (13.5)
Female	533 (45)
Indigenous	121 (10)
Comorbid conditions	
Diabetes mellitus	534 (49)
Hypertension	1079 (92)
Cardiovascular disease	288 (25)
Laboratory parameters	
CKD-EPI eGFR, ml/min per 1.73 m^2^	
Mean (SD)	34 (12)
Median (IQR)	33 (18)
30–59	708 (60)
15–29	412 (35)
10–14	55 (5)
UACR, mg/mmol	
Mean (SD)	71.5 (136.9)
Median (IQR)	12 (66.7)
<30	745 (63%)
≥30	428 (37%)
Outcomes	
KRT	109 (9)
Time to KRT, mo	
Mean (SD)	48 (40.5)
Median (IQR)	40 (44)
Within 2 yrs	30 (28)
Within 5 yrs	79 (72)
Death	
Total	214 (19)
Death after KRT start	27 (13)
Death without KRT	187 (87)

CKD-EPI, Chronic Kidney Disease Epidemiology Collaboration equation; eGFR, estimated glomerular filtration rate; IQR, interquartile range; KRT, kidney replacement therapy; UACR, urine albumin-to-creatinine ratio.

### KFRE Versus eGFR for Referral and KRT Outcomes

When the KFRE was applied to the total sample at 3% threshold over 5 years, 779 participants (66%) were identified as high risk for progressing to kidney failure requiring Kidney Replacement Therapy (KRT) Supplememntary Table S1. Of these 106 (14%) progressed to KRT, compared with 3 (1%) of those who were identified as low risk. In contrast, 466 participants (40%) fulfilled KHA’s eGFR threshold of < 30 ml/min per 1.73 m^2^ for specialist referral, with 72 (15%) progressing to KRT (Supplementary Table S2). Applying the KFRE to the sample of participants who did not fulfill the eGFR threshold for referral (*n* = 707) identified 330 (47%) as high risk, with 34 (10%) progressing to KRT, compared with 3 (1%) of those who were low risk (Supplementary Table S3).

### KRT Risk: 3% KFRE Versus 5% KFRE

In Table 2, we show the comparison of risk and KRT outcomes between the 3% and the 5% KFRE threshold over 5 years. When the 5% KFRE risk threshold was applied to the total sample of participants, 663 participants (57%) were deemed high risk for progression, compared with 779 (66%) when the 3% threshold was used (i.e., *n* = 116 fewer referrals). Applying the 5% KFRE threshold to participants who failed to fulfill the KHA eGFR threshold for referral identified 244 (34%) as high risk, compared with 330 (47%) (Table 2) when the 3% threshold was used. Of the 663 who were deemed high risk when the 5% KFRE threshold was used, 100 (15%) went on to require KRT, compared with 106 (10%) of those who were deemed high risk when the 3% KFRE threshold was used. If the 5% KFRE risk threshold replaced the eGFR threshold of 30ml/min per 1.73 m^2^ for referral, 25% of the participants would need reclassification, compared with 29% if the 3% KFRE threshold was used. (Table 2)

**Table 2:** KRT outcomes according to KFRE and eGFR thresholds

Parameter	3% KFRE	5% KFRE	eGFR < 30
5-yr risk on total sample	779 (66%)	663 (57%)	465 (40%)
5-yr risk on eGFR ≥ 30	330 (47%)	244 (34%)	
5-yr risk on eGFR < 30	449 (97%)	418 (90%)	
Number to KRT – total sample	106 (10%)	100 (15%)	
Number KRT - eGFR ≥ 30	34 (10%)	29 (12%)	
Number KRT - eGFR < 30	72 (16%)	71 (17%)	72 (15%)
Reclassification in either direction (KFRE + eGFR)	346 (43%)	291 (41%)	
Reclassification if KFRE replaced eGFR	29%	25%	

eGFR, estimated glomerular filtration rate; KFRE, kidney failure risk equation; KRT, kidney replacement therapy.

## Discussion

Our study found that the KFRE gave a good separation of risk in the total sample. However, more importantly, it provided a clearer separation of risk in participants who did not meet the KHA’s eGFR criteria for specialist referral, thus separating those who were likely to progress to require KRT from those who did not. When KFRE was applied to the whole sample, 779 participants (66%) were deemed high risk for progression and of these, 14% progressed to KRT, compared with < 1% of those who were in the low-risk group. When the KFRE was applied to the subgroup of participants who did not meet the KHA criteria for referral, 10% in the high-risk group went on to require KRT, compared with < 1% in the low-risk group, showing that even among participants who did not meet the KHA criteria for referral, KFRE was more sensitive to identifying participants at risk of progressing to KRT. The KFRE can therefore predict high risk even in participants who do not meet the KHA criteria while identifying those who are at low risk for progression; these are the group who could potentially be safely managed in primary care.

Different thresholds have been applied to evaluate the impact of the KFRE on referral patterns^6^, ^7^, ^8^, ^9^ and there does not appear to be consensus on the kidney failure risk threshold at which patients should be referred to a nephrologist. In our analysis, we applied the 3% and 5% risk thresholds over 5 years. Although the 5% threshold predicted less risk in the total sample (57% vs. 66%) and in the sample of participants who did not fulfill the eGFR threshold for referral (34% vs. 47%), it was more sensitive to predicting the progression to KRT both on the total sample (15% vs. 10%) and in the sample of participants who did not fulfill the eGFR threshold for referral (12% vs. 10%), giving a better association between predicted 5-year risk and observed KRT within the next 5 years. Furthermore, if the 5% KFRE risk replaced the eGFR threshold, there would be less reclassification of referrals than if the KFRE at 3% risk replaced the eGFR threshold (25% vs. 29%), suggesting that the application of the more stringent threshold of > 5% would identify fewer patients at high risk, hence resulting in fewer referrals to nephrology services.

The strength of our study was that our sample had a high completion rate of urine albumin-to-creatinine ratio measurement, with 68% of the participants having had a measured urine albumin-to-creatinine ratio within 12 months of a recorded eGFR, with 30% of participants having either a measured urine protein-to-creatinine ratio or urine dipstick, which allowed us to build a sizeable cohort for our analysis. The major limitation of our study is its retrospective nature; therefore, is likely to suffer the fundamental limitations that are commonly found in observational studies, such as potential confounding and selection bias.

In summary, our study demonstrated that the application of the KFRE establishes a clear separation of risk in individuals with stage G3 to G5 CKD, including those who do not meet the eGFR threshold for specialist referral. It also identified those who are at low risk for progression, who could be safely managed in primary care.

## Disclosure

All the authors declared no competing interests.

## Supplementary Material

Supplementary File (PDF) **Supplementary Methods. Supplementary References. Figure S1.** Study flow diagram. **Table S1.** Application of the KFRE to the whole sample. **Table S2.** Participants who fulfilled KHA eGFR threshold for referral. **Table S3.** Application of the KFRE to participants who did not fulfill the KHA criteria. **Table S4.** Referral criteria: 3%KFRE vs eGFR. **Table S5.** Referral based on eGFR criteria vs 3% KFRE.

## Footnotes

Supplementary File (PDF)

**Supplementary Methods.**

**Supple** **mentary References.**
**Figure S1.** Study flow diagram. **Table S1.** Application of the KFRE to the whole sample. **Table S2.** Participants who fulfilled KHA eGFR threshold for referral. **Table S3.** Application of the KFRE to participants who did not fulfill the KHA criteria.
