# Supplementary material for: Risk Stratification for CKD Progression in Primary Care
Source: Kidney Int Rep. 2025 Mar 6;10(5):1559–61. doi: 10.1016/j.ekir.2025.02.037 (PMC12142591; doi:10.1016/j.ekir.2025.02.037)
Supplement: Supplementary File (PDF) — Supplementary Methods. Supplementary References. Figure S1. Study flow diagram. Table S1. Application of the KFRE to the whole sample. Table S2. Participants who fulfilled KHA eGFR threshold for referral. Table S3. Application of the KFRE to participants who did not fulfill the KHA criteria. Table S4. Referral criteria: 3%KFRE vs eGFR. Table S5. Referral based on eGFR criteria vs 3% KFRE. [file mmc1.pdf]

Supplementary Material

Supplementary Introduction

KFRE thresholds

Different thresholds have been applied to evaluate the impact of the KFRE on referral patterns and there doesn't appear to be consensus on the kidney failure risk threshold at which patients should be referred to a nephrologist. However, whether 5% or 3% threshold is used, studies have demonstrated among other benefits, a reduction in unnecessary referrals and substantial reallocation of patients between primary care and specialist nephrology care(S1-S4). In our study we also applied the 3% and 5% risk thresholds over 5 years and assessed their impact on referrals compared to eGFR threshold.

Supplementary Methods

Patient Selection and Data Extraction

In this retrospective cohort study, we identified consecutive individual participants aged 18 years and above with Stage G3 to G5 CKD (eGFR 10 to 59ml/min/1.73m^2) who were enrolled into the CKD.QLD Registry. The CKD.QLD Registry is a collaborative of most public sector nephrology practices in Queensland (QLD) Australia. The main objective of the Registry was to profile all consenting participants with CKD, laying a foundation for CKD surveillance, practice improvement and research. The CKD.QLD data collection methods have previously been described by Venuthurapalli et. al(S5). Briefly, the Registry was designed to use a data linkage framework which consolidates data captured by multiple mechanisms into a single participant record, linked by a unique identifier. Participants already on KRT and those with acute kidney injury (AKI) were excluded unless they subsequently developed and met the diagnostic criteria for CKD. After commencing in May 2011, recruitment of new participants to the Registry was discontinued in May 2019.

For this study, participants were restricted to a single referral hospital due to their completeness of data items required for analysis. The aetiology of CKD in this cohort was previously described by Venuthurapalli et. al, showing diabetic nephropathy as the leading cause, followed by renovascular disease including hypertension and glomerulonephritis respectively, with unknown aetiology accounting for the smallest proportion of participants

(S6). A total of 1,520 participants enrolled between May 2011 and June 2018 were considered for analysis. Each participant's CKD.QLD identification number was matched with the corresponding QLD hospital record number. Demographic and clinical data were collected from all consenting adult participants with the diagnosis of CKD who presented to the public ambulatory nephrology clinic of the referral hospital. Additional data for analysis were captured incrementally in various formats, including: The Viewer (a read-only web-based application that displays consolidated clinical information for each participant, sourced from a number of existing Queensland Health (QH) enterprise clinical and administrative systems); Pathology QLD (a hierarchical networked system of 34 laboratories which provides pathology services to all QH public hospitals); and various other unit specific data capture mechanisms. Some of the missing data for laboratory parameters were directly collected from the two main private laboratory services in Qld, Sullivan Nicolaides Pathology and Queensland Medical Laboratory.

Three hundred and twenty-five participants with eGFR of less than 10ml/min or greater than 59ml/min at the time of referral were excluded, consistent with the original KFRE by Tangri et al (S7), whilst 22 participants did not have urine dipsticks, urine protein to creatinine ratio, or urine albumin to creatinine ratio. The final sample for analysis was 1, 173 participants.

Determination of Variables

The non-North American 4-variable KFRE was used to predict participants who will develop kidney failure within 5 years of referral to the nephrology clinic. The observed kidney failure rate obtained from the CKD.QLD Registry follow-up data and defined by commencement of KRT, was used as the reference. Variables accessible in the CKD.QLD database were integrated into the KFRE to calculate the proportion of participants who would fulfil criteria for referral at 5-year threshold of 3%. eGFR (calculated from serum creatinine using the CKD-EPI equation), uACR, gender and age retrieved from the Registry were used to calculate each participant's risk threshold for progression to kidney failure. Where only urine protein to creatinine ratio (uPCR) and dipstick protein were available, the equations developed by Sumida et al. (S8) were employed to calculate the predicted uACR. Where no uACR, uPCR or urine dipsticks were available at the initial visit, we used the earliest interval where the first urine protein examination was performed within the first six months of the initial visit in either direction. This increased the proportion of participants who had measured uACR available, given that most people seen in the nephrology clinics are

generally brought back for review within 3-6 months and that most of them would have urine examination for proteinuria/albuminuria ordered by the nephrologist. The date of the proteinuria/albuminuria measurement was used as the date for estimating baseline risk using the KFRE and for beginning the follow-up period.

Data Analysis

Descriptive statistics and basic inferential statistics were used to present participant's demographic and clinical characteristics. The number of participants in the database who met the Kidney Health Australia (KHA)'s eGFR criteria of $< 30ml/min/1.73m^2$ for nephrology referral was calculated. The KFRE was applied to all participants data to calculate the 5-year risk of progressing to kidney failure using a 3% KFRE risk threshold to stratify them into either high risk or low risk. We then applied the KFRE to the subgroup of participants who did not meet the KHA eGFR criteria for referral and compared the observed risk for progression to KRT to the predicted risk using a 3% risk threshold over 5 years. Participants whose 5-year risk for kidney failure was less than 3% were deemed low risk and hence could potentially have been safely managed in primary care, whereas all those with a 5-year risk of ≥3% would be classified as high risk and therefore would have been considered for a nephrology referral. We also evaluated the impact of applying the KFRE to the sample using a 5% risk threshold for kidney failure over 5 years and the 3% threshold in comparison to the KHA's eGFR threshold for specialist referral.

Supplementary References

S1. Major RW, Shepherd D, Medcalf JF, Xu G, Gray LJ, Brunskill NJ. The kidney failure risk equation for prediction of end stage renal disease in UK primary care: an external validation and clinical impact projection cohort study. PLoS Med. 2019;16(11):e1002955.

S2. Bhachu HK, Cockwell P, Subramanian A, Adderley NJ, Gokhale K, Fenton A, et al. Impact of using risk-based stratification on referral of patients with chronic kidney disease from primary care to specialist care in the United Kingdom. Kidney Int Rep. 2021;6(8):2189-99.

S3. Hingwala J, Wojciechowski P, Hiebert B, Bueti J, Rigatto C, Komenda P, et al. Risk-based triage for nephrology referrals using the kidney failure risk equation. Can J Kidney Health Dis. 2017;4:2054358117722782.

S4. Duggal V, Montez-Rath ME, Thomas IC, Goldstein MK, Tamura MK. Nephrology referral based on laboratory values, kidney failure risk, or both: a study using veterans affairs health system data. Am J Kidney Dis. 2021.

S5. Venuthurupalli SK, Hoy WE, Healy HG, Cameron A, Fassett RG. CKD.QLD: Establishment of a chronic kidney disease [CKD] registry in Queensland, Australia. BMC Nephrol. 2017;18(1):1-10. DOI: 10.1186/s12882-017-0607-5.

S6. Venuthurupalli SK, Healy H, Fassett R, Cameron A, Wang Z, Hoy WE. Chronic kidney disease, Queensland: Profile of patients with chronic kidney disease from regional Queensland, Australia: A registry report. Nephrology. 2019;24(12):1257-64.

S7. Tangri N, Stevens LA, Griffith J, Tighiouart H, Djurdjev O, Naimark D, et al. A predictive model for progression of chronic kidney disease to kidney failure. JAMA. 2011;305(15):1553-9. DOI: 10.1001/jama.2011.451.

S8. Sumida K, Nadkarni GN, Grams ME, Sang Y, Ballew SH, Coresh J, et al. Conversion of urine protein-creatinine ratio or urine dipstick protein to urine albumin-creatinine ratio for use in chronic kidney disease screening and prognosis: an individual participant-based meta-analysis. Ann Intern Med. 2020. DOI: 10.7326/M20-0529.

Supplementary Figure

Figure S1

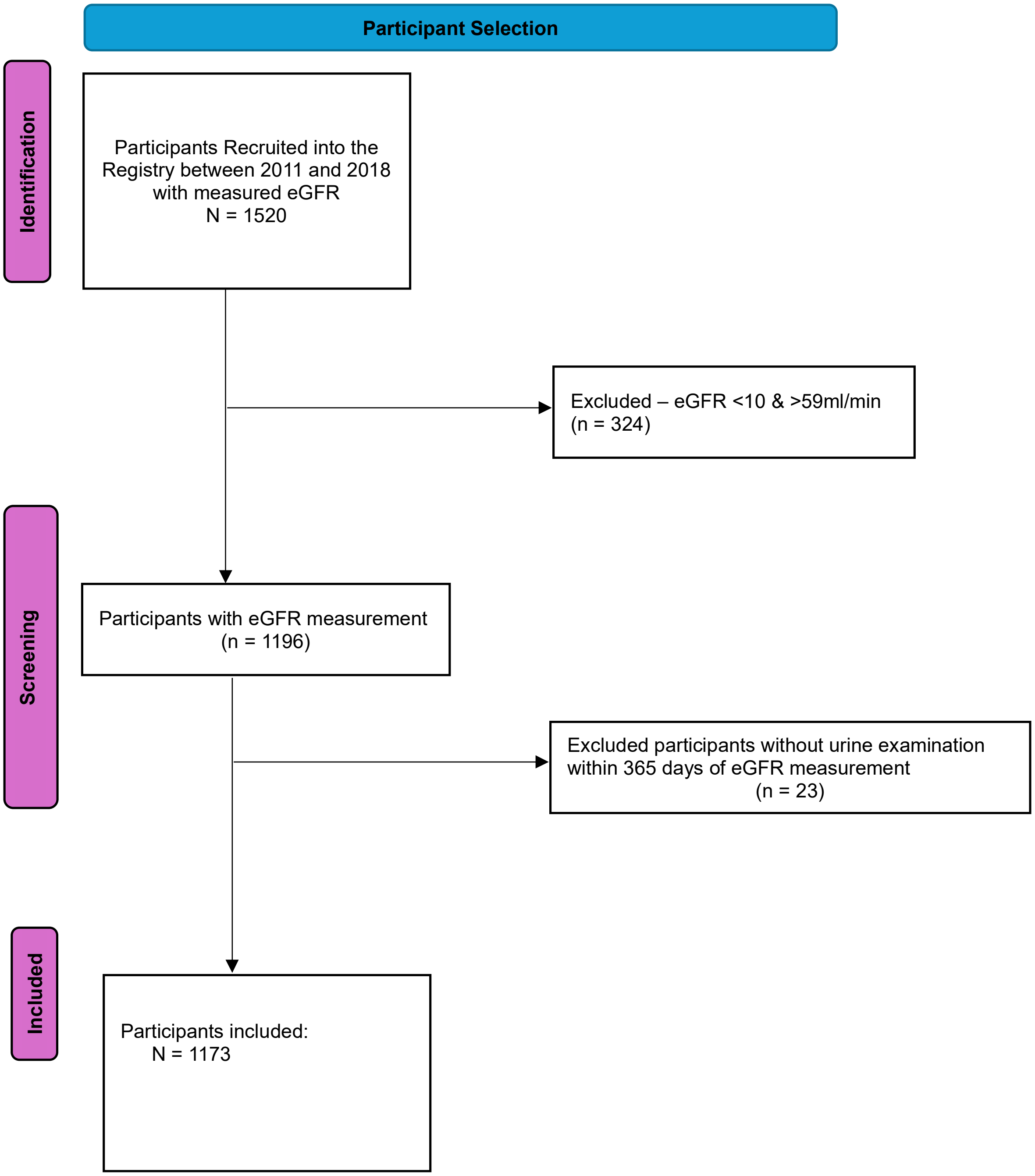

eGFR: Estimated glomerular filtration rate.

Supplementary Tables

Table S1 Application of the KFRE to the whole sample

KFRE	N = 1173 (%)	KRT (%)
KFRE ≥3%	779 (66%)	106 (14%)
KFRE <3%	394 (34%)	3 (0.8%)

KFRE, kidney failure risk equation; KRT, kidney replacement therapy.

Table S2 Participants who fulfilled KHA eGFR threshold for referral

KHA	N = 1173 (%)	KRT: n=109 (%)
eGFR <30	466 (40%)	72 (15%)
eGFR ≥30	707 (60%)	37 (5%)

KHA, kidney health Australia; KRT, kidney replacement therapy, eGFR, estimated glomerular filtration rate.

Table S3: Application of the KFRE to participants who did not fulfill the KHA criteria

KFRE	N = 707	KRT
KFRE ≥3%	330 (47%)	34 (10%)
KFRE <3%	377 (53%)	3 (0.8%)

KFRE, kidney failure risk equation; KRT, kidney replacement therapy
